# Use of multiple inflammatory marker tests in primary care: using Clinical Practice Research Datalink to evaluate accuracy

**DOI:** 10.3399/bjgp19X704309

**Published:** 2019-06-18

**Authors:** Jessica Watson, Hayley E Jones, Jonathan Banks, Penny Whiting, Chris Salisbury, Willie Hamilton

**Affiliations:** Population Health Sciences, Bristol Medical School, University of Bristol, Bristol.; Population Health Sciences, Bristol Medical School, University of Bristol, Bristol.; Population Health Sciences, Bristol Medical School, University of Bristol, Bristol.; Population Health Sciences, Bristol Medical School, University of Bristol, Bristol.; Population Health Sciences, Bristol Medical School, University of Bristol, Bristol.; University of Exeter Medical School, University of Exeter, Exeter.

**Keywords:** blood plasma, blood tests, c-reactive protein, erythrocyte sedimentation rate, primary care

## Abstract

**Background:**

Research comparing C-reactive protein (CRP), erythrocyte sedimentation rate (ESR), and plasma viscosity (PV) in primary care is lacking. Clinicians often test multiple inflammatory markers, leading to concerns about overuse.

**Aim:**

To compare the diagnostic accuracies of CRP, ESR, and PV, and to evaluate whether measuring two inflammatory markers increases accuracy.

**Design and setting:**

Prospective cohort study in UK primary care using the Clinical Practice Research Datalink.

**Method:**

The authors compared diagnostic test performance of inflammatory markers, singly and paired, for relevant disease, defined as any infections, autoimmune conditions, or cancers. For each of the three tests (CRP, ESR, and PV), sensitivity, specificity, positive predictive value (PPV), negative predictive value (NPV), and area under receiver operator curve (AUC) were calculated.

**Results:**

Participants comprised 136 961 patients with inflammatory marker testing in 2014; 83 761 (61.2%) had a single inflammatory marker at the index date, and 53 200 (38.8%) had multiple inflammatory markers. For *‘any relevant disease’*, small differences were seen between the three tests; AUC ranged from 0.659 to 0.682. CRP had the highest overall AUC, largely because of marginally superior performance in infection (AUC CRP 0.617, versus ESR 0.589, *P*<0.001). Adding a second test gave limited improvement in the AUC for relevant disease (CRP 0.682, versus CRP plus ESR 0.688, *P*<0.001); this is of debatable clinical significance. The NPV for any single inflammatory marker was 94% compared with 94.1% for multiple negative tests.

**Conclusion:**

Testing multiple inflammatory markers simultaneously does not increase ability to rule out disease and should generally be avoided. CRP has marginally superior diagnostic accuracy for infections, and is equivalent for autoimmune conditions and cancers, so should generally be the first-line test.

## INTRODUCTION

Inflammatory markers, including C-reactive protein (CRP), erythrocyte sedimentation rate (ESR), and plasma viscosity (PV) are commonly used in primary care for diagnosis and monitoring of inflammatory conditions, including infections, autoimmune conditions, and cancers.^1^ Rates of inflammatory marker testing are rising, with a consistent linear increase in testing rates for CRP over the past 15 years.^2^ There is significant variation between GP practices in inflammatory marker testing rates and frequency of abnormal results.^3^

Erythrocyte sedimentation rate is the oldest of the three inflammatory markers, defined as the distance in millimetres that erythrocytes settle in anticoagulated whole blood in 1 hour. Plasma viscosity is generally considered to be superior to ESR, being unaffected by age, sex, anaemia, or polycythaemia, all of which may influence ESR. C-reactive protein is often thought to be superior for infections, rising more rapidly than the other two tests in response to inflammation. However, little research compares these three tests,^4^ and clinicians report uncertainty around their optimum usage.^5^ Furthermore, clinicians often test multiple inflammatory markers simultaneously, leading to concerns about overuse. This is particularly important as research has shown that false-positives are common after inflammatory marker testing, and can lead to increased follow-on GP appointments, tests, and referrals.^6^

The aim of this study was to compare the accuracy of using single inflammatory marker tests versus multiple inflammatory marker tests in combination.

## METHOD

Methods by the authors have been described fully previously.^6^ A total of 160 000 patient participants from the Clinical Practice Research Datalink (CPRD) who had inflammatory marker testing in 2014 were initially selected. The index date was defined as the first date of inflammatory marker testing in 2014. All analyses reported are based on the inflammatory marker test or tests carried out simultaneously on the index date; sequential testing was not examined. Participants with a pre-existing diagnosis of cancer or autoimmune conditions in the 2 years before the index date were excluded, as were patients with an acute infection in the 30 days before the index date.

The primary outcome was *‘any relevant disease’*, defined as any autoimmune condition or cancer coded within 1 year of the index date, or infection within 1 month of the index date. Code lists were developed using published methods^7^ and are available on the University of Bristol Data Repository.^8^ The researchers used previously developed code lists (available from the authors on request) as well as linked data from the English Cancer Registry for cancer codes.

**How this fits in**

There is a lack of research comparing the accuracy of inflammatory markers. Testing multiple inflammatory markers is common, leading to concerns about overuse. In this large observational study using UK primary care electronic health records the authors found very little difference between the accuracy of C-reactive protein (CRP), erythrocyte sedimentation rate (ESR), and plasma viscosity (PV). CRP had slightly superior diagnostic accuracy for infections, and was equivalent for autoimmune conditions and cancers; the authors therefore suggest this should be the first-line test in most circumstances. Testing multiple inflammatory markers does not increase the ability to rule out disease and should generally be avoided.

### Index tests

The index tests were CRP, ESR, and PV; test results were dichotomised into raised or normal using the mean upper limit of normal from laboratories within this study (>7 mg/L for CRP; >1.72 mm/hour for PV; upper limits of normal, stratified by age and sex, for ESR are available from the authors on request). A binary variable ‘any raised inflammatory marker’ was generated if any of CRP, PV, or ESR were raised.

### Accuracy of CRP, PV, and ESR as single tests

For each of the three tests (CRP, ESR, and PV), dichotomised test results were cross-classified with the reference standard *‘any relevant disease’,* allowing sensitivity, specificity, positive predictive value (PPV), and negative predictive value (NPV) to be calculated. Logistic regression was used to calculate diagnostic odds ratios, with and without adjustment for age and sex.

To address potential concerns that differences in patient mix could lead to biased estimates, for example, CRP used preferentially in patients with suspected infection, sensitivity analyses were conducted on the subgroup with two tests performed simultaneously to allow head-to-head comparison of diagnostic test accuracy.

Test results were also treated as continuous variables on a log scale, owing to their skewed distribution, to assess their predictive value in a logistic regression model, including age and sex as additional explanatory variables, calculating the area under curve (AUC). The AUCs for CRP versus ESR plus CRP versus PV were compared using the DeLong method,^9^ generating confidence intervals (CIs) and *P*-values. Sub-analyses compared AUCs for disease subtypes, including infections, autoimmune conditions, and cancers.

### Accuracy of test results in combination

**Figure 1.**
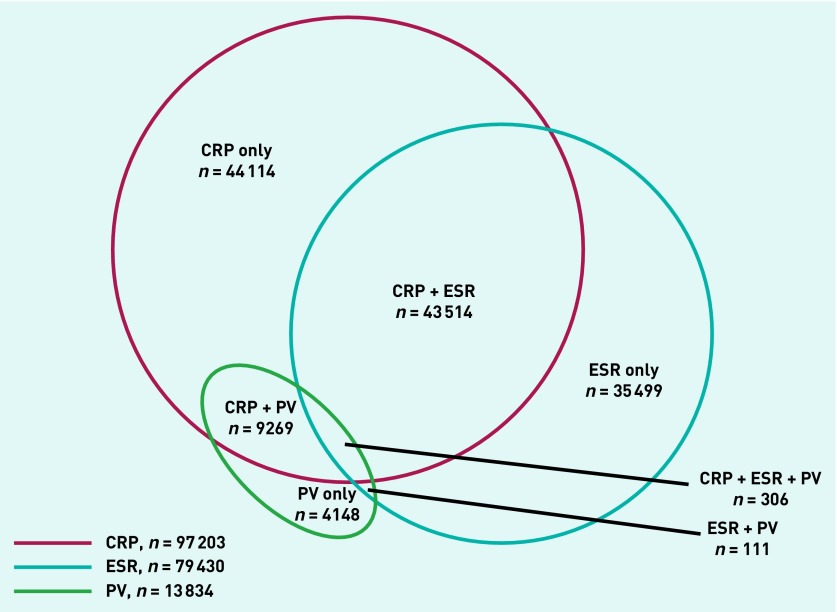
***Inflammatory marker tests requested. CRP = C-reactive protein. ESR = erythrocyte sedimentation rate. PV = plasma viscosity.***

The authors examined the accuracy of two combinations of inflammatory markers: CPR plus ESR and CRP plus PV. Only 111 patients had ESR plus PV and 306 had all three tests (Figure 1), therefore the researchers did not examine these test combinations. Where two inflammatory marker tests were performed simultaneously, measures of diagnostic accuracy were calculated (sensitivity/specificity/PPV/NPV) for two alternative definitions of an overall positive result:

- both inflammatory markers raised (denoted, for example, as CRP + ESR or CRP + PV)
  – defined as a combined test where both inflammatory markers tested were positive; and
- either inflammatory marker raised (denoted, for example, as CRP|ESR or CRP|PV)
  – defined as a combined test where either of the inflammatory markers tested were positive.

The AUC for test combinations were calculated using a logistic regression model with log-transformed test values, including age and sex as covariates. An interaction term was used in the model due to the associations between inflammatory marker test results. All analyses were carried out using Stata (version 15).

## RESULTS

From 12 905 266 potentially eligible patients aged >18 years found in CPRD GOLD in 2014, 46 3304 (3.6%) had at least one inflammatory marker test in 2014. Of these, 160 000 were selected at random by CPRD. Out of these, 22 377 with pre-existing disease were excluded (3692 cancers, 10 427 autoimmune disease, 8258 infections) as well as 660 with missing test results and two with test results so abnormal they were considered spurious, leaving 136 961 in the final cohort. The cohort was 61.6% female with a median age of 55.4 years (interquartile range [IQR] 41.1–69.9 years).

### Tests requested

Figure 1 shows the tests requested; 71.0% (*n* = 97 203) had a CRP test, 58.0% had an ESR (*n* = 79 430), and 10.1% (*n* = 13 834) had a PV test. Of those tested, 83 761 (61.2%) had a single inflammatory marker tested at the index date, and 53 200 (38.8%) had >1 inflammatory marker; mostly CRP plus ESR (*n* = 43 514), followed by CRP and PV (*n* = 9269). A lower number, *n* = 306, had all three inflammatory markers tested simultaneously. The single inflammatory marker group was 61.0% female with a median age of 56.0 years (IQR 41.5–70.8 years) compared with 62.5% female, median age 54.5 years (IQR 40.6–68.6 years) in the multiple tested group.

### Test results and overall disease incidence

In the single test group 23.8% (*n* = 19 932) had a raised inflammatory marker (Table 1). In comparison, 34.0% (*n* = 18 078) of the multiple test group had one or more raised inflammatory marker; 12.8% (*n* = 6803) had concordant raised values and 21.2% (*n* = 11 275) had discordant results (one raised, one normal). Overall disease incidence was 8.2% in the single tested group compared with 9.0% in the multiple tested group. Furthermore, in the multiple tested group, disease incidence was higher in the group with concordant raised values (22.6%, *n* = 1539), compared with those with a single raised value (10.4% *n* = 1174). The NPV for any single normal inflammatory marker was 94%, compared with 94.1% with multiple normal tests (Table 1 footnotes a and c).

**Table 1.** 2×2 table of test results and overall disease incidence for single-versus double-tested patients, *N* = 136 961

**Test result**	**Disease positive, *n* (%)**	**Disease negative, *n* (%)**	**Total, *n* (%)**
**Single inflammatory marker, *N* = 83 761**			
Raised	2999 (15.0)	16 933 (85.0)	19 932 (23.8)
Normal	3847 (6.0)	59 982 (94.0)^a^	63 829 (76.2)

**Multiple inflammatory markers,^b^*N*= 53 200**			
Multiple raised inflammatory markers	1539 (22.6)	5264 (77.4)	6803 (12.8)
Discordant results	1174 (10.4)	10 101 (89.6)	11 275 (21.2)
All normal	2065 (5.9)	33 057 (94.1)^c^	35 122 (66.0)

a Negative predictive value of a single normal inflammatory marker.

b Includes patients with two and three inflammatory marker tests. If two or more tests were raised patients were included in the ‘multiple raised inflammatory markers’; those with only one raised inflammatory marker were included in the ‘discordant results’ category.

c Negative predictive value of multiple normal inflammatory markers.

### Comparative accuracy of CRP, ESR, and PV as single tests

C-reactive protein, ESR, and PV were broadly similar in terms of sensitivity, specificity, PPV, and NPV (Table 2). Measures of diagnostic accuracy were relatively unchanged when analysis was limited to subgroups with two inflammatory markers performed simultaneously (additional analyses available from the authors on request). Two pairwise comparisons of AUC for CRP versus ESR (Table 3) and CRP versus PV (Table 4) in those with multiple simultaneous tests were completed. For ‘any relevant disease’, small differences were seen between the three tests; with AUC ranging from 0.659 to 0.682. Very few patients had ESR and PV tests simultaneously, so the researchers were not able to directly compare these two tests.

**Table 2.** Comparison of measures of diagnostic accuracy for CRP, ESR, and PV, singly and in combination for diagnosis of any relevant disease (infection, autoimmune condition, or cancer)

**Test and test combinations**	**Patients, *n***	**True-positive, *n* (%)**	**False-positive, *n* (%)**	**True-negative, *n* (%)**	**False-negative, *n* (%)**	**Sensitivity (95% CI)**	**Specificity (95% CI)**	**PPV (95% CI)**	**NPV (95% CI)**	**DOR unadjusted (95% CI)**	**DOR adjusted^a^ (95% CI)**
CRP	97 203	3947 (4.1)	18 745 (19.3)	69 797 (71.8)	4714 (4.8)	45.6 (44.5 to 46.6)	78.8 (78.6 to 79.1)	17.4 (16.9 to 17.9)	93.7 (93.5 to 93.9)	3.12 (2.98 to 3.26)	2.86 (2.73 to 2.99)
ESR	79 430	2780 (3.5)	15 589 (19.6)	57 221 (72.0)	3840 (4.8)	42.0 (40.8 to 43.2)	78.6 (78.3 to 78.9)	15.1 (14.6 to 15.7)	93.7 (93.5 to 93.9)	2.66 (2.52 to 2.80)	2.43 (2.30 to 2.55)
PV	13 834	536 (3.9)	3242 (23.4)	9439 (68.2)	617 (4.5)	46.5 (43.6 to 49.4)	74.4 (73.7 to 75.2)	14.2 (13.1 to 15.3)	93.9 (93.4 to 94.3)	2.53 (2.24 to 2.86)	2.32 (2.05 to 2.62)
CRP + ESR^b^	43 820	1277 (2.9)	4269 (9.7)	35 574 (81.2)	2700 (6.2)	32.1 (30.7 to 33.6)	89.3 (89.0 to 89.6)	23.0 (21.9 to 24.2)	93.0 (92.7 to 93.2)	3.94 (3.66 to 4.24)	3.56 (3.31 to 3.84)
CRP|ESR^c^	43 820	2225 (5.1)	12 271 (28.0)	27 572 (62.9)	1752 (4.0)	56.0 (54.4 to 57.5)	69.2 (68.8 to 69.7)	15.4 (14.8 to 16.0)	94.0 (93.8 to 94.3)	2.85 (2.67 to 3.05)	2.61 (2.44 to 2.79)
CRP + PV	9575	267 (2.8)	974 (10.2)	7784 (81.3)	550 (5.7)	32.7 (29.5 to 36.0)	88.9 (88.2 to 89.5)	21.5 (19.3 to 23.9)	93.4 (92.9 to 93.9)	3.88 (3.30 to 4.56)	3.56 (3.02 to 4.19)
CRP|PV	9575	495 (5.2)	3110 (32.5)	5648 (59.0)	322 (3.4)	60.6 (57.1 to 64.0)	64.5 (63.5 to 65.5)	13.7 (12.6 to 14.9)	94.6 (94.0 to 95.2)	2.79 (2.41 to 3.23)	2.59 (2.24 to 3.01)

a DOR = diagnostic odds ratio, adjusted for age and sex.

b CRP + ESR; positive test defined as both CRP and ESR positive.

c CRP|ESR; positive test defined as either CRP or ESR positive. CRP = C-reactive protein. ESR = erythrocyte sedimentation rate. NPV = negative predictive value. PPV = positive predictive value. PV = plasma viscosity.

**Table 3.** Comparison of overall test performance among those with both CRP and ESR performed simultaneously, *N* = 43 820^a^

**Disease outcome^b^**	***n***	**CRP AUC^c^ (95% CI)**	**ESR AUC (95% CI)**	***P*-value for CRP versus ESR**	**CRP and ESR AUC (95% CI)**	***P*-value for combined versus better single test**
Any relevant disease	3977	0.682 (0.672 to 0.690)	0.665 (0.656 to 0.674)	<0.001	0.688 (0.678 to 0.697)	<0.001
Infections	1565	0.617 (0.601 to 0.632)	0.589 (0.574 to 0.603)	<0.001	0.619 (0.604 to 0.634)	0.018
Autoimmune conditions	1663	0.710 (0.697 to 0.724)	0.708 (0.695 to 0.721)	0.680	0.724 (0.710 to 0.737)	<0.001
Cancer	882	0.774 (0.759 to 0.788)	0.766 (0.752 to 0.781)	0.017	0.777 (0.763 to 0.791)	0.006
Polymyalgia rheumatica/giant cell arteritis	476	0.882 (0.880 to 0.900)	0.872 (0.860 to 0.890)	0.099	0.887 (0.874 to 0.900)	0.110
Rheumatoid arthritis	557	0.691 (0.670 to 0.712)	0.690 (0.669 to 0.711)	0.890	0.700 (0.679 to 0.721)	0.007
Seronegative arthritis	151	0.700 (0.653 to 0.746)	0.686 (0.638 to 0.734)	0.510	0.706 (0.659 to 0.753)	0.540
Inflammatory bowel disease	223	0.698 (0.660 to 0.737)	0.691 (0.653 to 0.730)	0.450	0.701 (0.662 to 0.740)	0.510

a Only 9494 (total amount in 1st column) out of the total 43 820 had a relevant disease outcome. The analysis of test performance uses those with and without relevant disease hence the total n = 43 820 is correct here.

b Where disease subtypes were examined, diseases other than the specified condition reported were classified as non-diseased.

c AUC = area under the receiver operator curve, where AUC = 0.5 is equivalent to no diagnostic utility and AUC = 1 is perfect diagnostic accuracy. AUC was calculated using logistic regression modelling with test result(s) on a log scale and age and sex as additional explanatory variables. CRP = C-reactive protein. ESR = erythrocyte sedimentation rate.

On comparing CRP with ESR, it was found that CRP had a slightly higher AUC for infection (AUC 0.617, 95% CI = 0.601 to 0.632 versus 0.589, 95% CI = 0.574 to 0.603, *P*<0.001, Table 3). The authors found no significant difference in the AUC of CRP and ESR for diagnosis of autoimmune conditions, and no significant difference for the main subtypes of autoimmune disease: polymyalgia rheumatica, rheumatoid arthritis, seronegative arthritis, or inflammatory bowel disease.

On comparing CRP and PV it was found that CRP had a higher AUC for infection (AUC 0.638, 95% CI = 0.608 to 0.670 versus 0.597, 95% CI = 0.564 to 0.628, *P* = 0.004 [Table 4]), with no difference for cancer (*P* = 0.49) or autoimmune disease (*P* = 0.97). The authors did not compare the accuracy of CRP versus PV for subtypes of autoimmune disease due to the smaller sample size for PV tests.

**Table 4.** Comparison of overall test performance among those with both CRP and PV performed simultaneously, *N* = 9575

**Disease outcome^a^**	***n***	**CRP AUC^b^ (95% CI)**	**PV AUC (95% CI)**	***P*-value for CRP versus PV**	**CRP and PV AUC (95% CI)**	***P*-value for combined versus better single test**
Any relevant disease	817	0.672 (0.652 to 0.692)	0.659 (0.640 to 0.679)	0.170	0.686 (0.667 to 0.706)	0.004
Infection	325	0.638 (0.608 to 0.670)	0.597 (0.564 to 0.628)	0.004	0.639 (0.608 to 0.669)	0.280
Autoimmune conditions	338	0.687 (0.657 to 0.717)	0.686 (0.655 to 0.717)	0.970	0.709 (0.679 to 0.739)	0.009
Cancer	183	0.753 (0.720 to 0.787)	0.760 (0.726 to 0.793)	0.490	0.764 (0.731 to 0.797)	0.170

a Where disease subtypes were examined, diseases other than the specified condition reported were classified as non-diseased.

b AUC = area under the receiver operator curve, where AUC = 0.5 is equivalent to no diagnostic utility and AUC = 1 is perfect diagnostic accuracy. AUC was calculated using logistic regression modelling with test result(s) on a log scale and age and sex as additional explanatory variables. CRP = C-reactive protein. PV = plasma viscosity.

### Accuracy of test results in combination: sensitivity, specificity, PPV, and NPV

For two simultaneous tests, by definition, sensitivity and specificity vary depending on how the results are interpreted (Table 2). If an overall positive result was defined as *both* inflammatory markers raised, for example, CRP plus ESR, then PPVs were higher and specificity was increased, but at the price of lower sensitivity, compared with using any single test.

If the combined test was defined as *either* inflammatory marker raised, for example, CRP|ESR, then sensitivity increased but specificity fell compared with any single test. This led to fewer false-negatives or reduced risk of missed diagnoses but a markedly increased frequency of false-positives, for example, CRP alone generated false-positives in 19.3% of those tested, compared with 32.5% false-positives for CRP|PV (Table 2, column 4). The maximum sensitivity was 60.6% for the test combination CRP|PV (Table 2, column 7).

### Accuracy of test results in combination: area under curve (AUC)

The authors compared the accuracy of CRP and ESR in combination, compared with the better of the two individual tests (Table 3). Adding a second test gave limited improvement in the AUC for relevant disease (CRP 0.682, 95% CI = 0.672 to 0.690 versus CRP plus ESR 0.688, 95% CI = 0.678 to 0.697, *P*<0.001). There was no improvement in AUC for CRP and ESR in combination for infection. The combined test CRP plus ESR gave an increase of 0.014 in the AUC for autoimmune disease (*P*<0.001) and 0.003 increase in AUC for cancers (*P* = 0.006) compared with single CRP test. While this was statistically significant, it is unlikely to be of a magnitude to be clinically significant. The combined test did not increase the AUC for polymyalgia rheumatica, seronegative arthritis, or inflammatory bowel disease, and led to a small increase of 0.009 in the AUC for rheumatoid arthritis (*P* = 0.007).

Similarly, the combination of CRP and PV together gave no improvement in AUC, compared with the better of the two individual tests, for infection or cancer (Table 4). The combined test CRP and PV gave an increase of 0.022 in the AUC for autoimmune disease, which was statistically significant, but seems unlikely to be clinically significant.

## DISCUSSION

### Summary

In this large study of UK inflammatory marker testing, the authors found the practice of requesting multiple inflammatory markers to be remarkably common, perhaps reflecting increases in overall primary care testing rates.^2^ Multiple testing was associated with more abnormal and more discordant results. The authors found no evidence that this approach helps to rule out serious pathology, as the NPV of a single inflammatory marker (94.0%) was the same as the NPV of combined inflammatory markers (94.1%) (Table 1). Furthermore, discordant results may be challenging to interpret, critically, whether the clinician should regard one abnormal test as sufficient or should require both to be abnormal before further investigation or treatment. No combination of inflammatory marker tests can be used to rule in or rule out disease confidently. The maximum sensitivity of 60.6% (for the combined test CRP|PV) is low, yet comes at a price of increased false-positives compared with using single tests.

In diagnosis of infections, CRP marginally outperforms both ESR and PV. The three tests are equivalent for diagnosis of autoimmune diseases and cancers. Overall, inflammatory markers have a low AUC for most disease outcomes, with the exception of polymyalgia rheumatica. Testing multiple inflammatory markers, perhaps unsurprisingly, produces a higher PPV if both tests are raised (22.6%) compared with a single raised inflammatory marker (PPV 15.0%). This benefit is offset however by the low sensitivity once a double positive result is required (32.1% for CRP plus ESR and 32.7% for CRP plus PV), meaning that more pathology would be missed with this testing strategy, making it less helpful for ruling out disease. Testing two inflammatory markers does not appear to improve the overall discriminatory ability measured by the AUC. The small differences in AUC between single and double inflammatory marker tests for autoimmune conditions and cancer are probably of little clinical value, even if statistically significant.

Testing multiple inflammatory markers simultaneously does not improve the ability to rule out disease. It leads to increased rates of discordant results and increases costs without tangible benefits. CRP should generally be the first-line test, with the possible exception of myeloma. It must be remembered that all inflammatory markers have relatively poor performance characteristics, so perhaps is it no surprise that two tests are no better than one.

### Strengths and limitations

The major strengths of this study are its size and the setting in primary care, where the initial suspicion of disease usually arises. Given the large sample size, the researchers have been able to directly compare diagnostic accuracy in patients with two inflammatory markers performed simultaneously. This reduces the potential for selection bias, where tests might perform better for certain disease outcomes due to GPs pre-selecting those at higher risk to have a specific test, for example, preferentially using CRP when an infection is suspected. There the possibility remains that patients with multiple inflammatory markers may differ from those with a single test; this is reflected by the fact that overall rates of disease were 9.2% in the double-tested compared with 8.2% in the single-tested group. This may influence the generalisability of the present results; however, the finding that measures of diagnostic accuracy are very similar in sensitivity analyses limited to the double-tested groups suggests that this is a relatively minor effect. One complication of a large sample size is that statistically significant differences can be found that are of little clinical significance; the authors have tried to highlight where this occurred.

Using routine data for diagnostic accuracy studies rather than prospectively performing multiple tests and evaluating a single disease outcome more closely reflects the diagnostic dilemmas facing GPs; however, this innovative approach does bring inherent challenges. The authors chose to use 1-year incidence of cancer and autoimmune disease, and 1-month incidence of infection, as a proxy for prevalence of disease at the time of testing. This is a pragmatic choice, based on evidence of the time lag between symptomatic presentation and diagnosis of cancer,^10^ but, as a result, some of the diagnoses may be unrelated to the initial inflammatory marker test result.

All studies using electronic health records are reliant on the quality of data recording; however, blood tests are transferred electronically into the notes and diagnoses tend to be recorded with greater accuracy than symptoms.^11^ The authors also used cancer registry data to improve cancer outcome ascertainment. Diagnosis of infection is likely to be less well coded, and microbiological confirmation of diagnosis is rarely obtained, leading to potential biases.

In clinical practice several factors determine who is tested: the patient, the symptoms, and the GP. Though the researchers have data on the demographics of the patients tested, they do not know what symptoms triggered testing and therefore cannot determine which tests were done for specific diagnostic purposes, and which were done as a general rule-out test for any relevant underlying disease. Demographic characteristics of GPs may also influence the choice of inflammatory marker test used; however, the researchers did not have GP identifiers so were not able to explore potential clustering by GP.

The benefit of the present approach is that it reflects real-life clinical practice; though GPs may not have a specific diagnosis in mind when they request inflammatory markers, they need to consider a wide range of possible diagnoses if the test is positive.

### Comparison with existing literature

In a previous systematic review, limited evidence comparing CRP and ESR was found for a small number of specialist disease outcomes in secondary care settings.^4^ The limited evidence available prevented the authors from making recommendations about the preferred choice of test. The PPVs in this study are lower than those reported in that review; this is likely to reflect the low disease prevalence in the primary care setting.

UK guidelines for diagnosis of polymyalgia recommend measuring ESR and CRP;^12^ however, the authors were not able to demonstrate an improvement in diagnostic accuracy from combining these two tests. Previous studies have shown that inflammatory markers can sometimes be normal in both polymyalgia and giant cell arteritis;^13^^,^^14^ in another study, most of those with normal ESR had raised CRP.^15^ In cases of diagnostic uncertainty, repeat testing is often warranted, a different inflammatory marker may be added at this stage, or the same test repeated, expecting a change over time. The authors were unable to examine this.

Previous studies have shown that ESR and PV are superior to CRP for the diagnosis of myeloma.^16^ Due to the small number of myeloma cases in the present sample the researchers were unable to corroborate this finding.

Although the authors have been able to show moderate predictive value of inflammatory markers for inflammatory bowel disease (IBD), the AUC for CRP of 0.698 (in a model that includes age and sex) is much lower than for calprotectin with a published AUC of 0.95,^17^ therefore calprotectin is to be preferred if IBD is under consideration. Similarly, though inflammatory markers have a modest AUC for rheumatoid arthritis, low sensitivities found in the present study are in keeping with previous studies, which have found that 35% to 45% of patients with rheumatoid arthritis have normal inflammatory marker levels at diagnosis;^18^ National Institute for Health and Care Excellence guidelines therefore recommend referral of patients with clinical evidence of rheumatoid arthritis, even with normal inflammatory marker test results.^19^ It is therefore hard to see any benefits from inflammatory marker testing where rheumatoid arthritis is suspected diagnostically, though it may have a useful role in disease monitoring.

### Implications for practice

Testing multiple inflammatory markers does not improve the ability to rule out disease, but does increase the risk that at least one of the tests will give a false-positive, compared with a strategy of using a single test. The authors therefore suggest that this should generally be avoided, in keeping with primary care guidance in New Zealand.^20^

The overall diagnostic utility of all three inflammatory markers is similar and low, however CRP marginally outperforms ESR and PV for infections. CRP also tends to be cheaper than either ESR or PV (1.19 GBP for CRP, 3.18 GBP for ESR, 3.18 GBP for PV: source Bristol North Somerset and South Gloucester CCG laboratory costings). The authors therefore suggest that CRP should be the first-line test in most circumstances. Exceptions might include the use of ESR or PV rather than CRP for suspected myeloma (given that the authors have no evidence to support or refute previous findings), though if there is strong clinical suspicion then direct testing using electrophoresis and Bence Jones protein is preferable.

There is no combination of inflammatory markers that can be used as a reliable rule-in or rule-out test strategy. Results and decisions to test must be made in the context of other clinical findings. Faced with low probability of disease, for example, ‘low-risk-but-not-no-risk’ cancer symptoms, inflammatory markers may still offer some clinical utility. They should however be interpreted in a Bayesian manner, with a positive test result increasing disease likelihood, and a negative test reducing disease likelihood, with neither being definitive. However, a negative test in the clinical context of a low-likelihood situation may be sufficient to provide reassurance.
